# Transvenous biopsy of body cistyc lesions in a 32-year-old man with cloves syndrome and thrombocytopenia: a safe option for high bleeding risk patients

**DOI:** 10.1186/s42155-025-00541-2

**Published:** 2026-03-18

**Authors:** María Alejandra Martínez, Jose Rodrigo Muñoz-Ordoñez, Jorge Alberto Guzman, María Paula González‑Zambrano, Diego Alejandro Gomez Castellanos, María F. Estévez, Edgar Fabian Manrique‑Hernandez

**Affiliations:** 1 Present Address: Departamento de Radiología Intervencionista, Hospital Internacional De Colombia (HIC), Valle de Terrazas De Menzuly #Km 7, Piedecuesta, Santander Colombia; 2 Present Address: Universidad El Bosque, Bogotá, DC Colombia; 3 Fundación Oftalmológica de Santander, Clínica Ardila-Lülle, Floridablanca, Santander Colombia

## Abstract

**Introduction:**

Image guided percutaneous biopsy is a standard in interventional radiology but can be limited by anatomical or clinical factors. Alternative techniques, such as transvenous biopsy, can aid in cases with these constraints, particularly in patients with thrombocytopenia.

**Case presentation:**

A 32-year-old male with PIK3CA related overgrowth syndrome presenting as Congenital, Lipomatous, Overgrowth, Vascular malformations, Epidermal nevi, Spinal/skeletal anomalies and/or scoliosis (CLOVES) syndrome, was admitted to the emergency department with bleeding cystic lesions and severe thrombocytopenia. The initial medical approach for patients with thrombocytopenia must be supported by a genetic or histopathologic examination, as required by insurance protocols. Percutaneous fine-needle biopsy often has low diagnostic yield in such cases. Due to the high risk of bleeding associated with thrombocytopenia and the vascular nature of the cystic lesions, direct percutaneous biopsy was contraindicated. Instead, a transvenous biopsy was performed by accessing the lesion through a venous route under image guidance, allowing for safe tissue sampling without the risk of significant hemorrhage. This approach confirmed PIK3CA involvement and guided subsequent treatment.

**Conclusions:**

Transvenous biopsy serves as a safe and effective alternative to standard percutaneous biopsy in high-risk patients with thrombocytopenia and vascular lesions, enabling accurate diagnosis while minimizing bleeding complications.

## Introduction

Image guided percutaneous biopsy has become one of the most common and rapidly increasing procedures in interventional radiology [1]. This approach plays a crucial role in the diagnosis and management of various diseases. However, its application is not always optimal or straightforward. Anatomical factors such as the presence of important structures like intestinal loops, major blood vessels, bones, and lungs, as well as the patient’s body mass index, can hinder access to the biopsy site. Another important factor is the clinical condition in which the procedure is not feasible, such as in patients with thrombocytopenia or coagulation disorders, which motivates the exploration of alternative pathways for obtaining tissue samples [1, 2].

Today, many treatments and therapies for different diseases are based on the need for genetic isolation, as well as genomic studies and biomarkers that help assess disease progression and prognosis in each patient. This facilitates the identification of various appropriate therapeutic options [3, 4]. This case report highlights the potential of transvenous biopsy as a viable option in scenarios where percutaneous biopsies are contraindicated, Demonstrating its utility as a safe vascular access technique for high-risk patients who are unsuitable candidates for conventional biopsy methods [1, 2, 5].

## Case presentation

PIK3CA-related overgrowth spectrum (PROS) encompasses a group of rare disorders caused by somatic mutations in the PIK3CA gene, which encodes the p110α catalytic subunit of phosphatidylinositol 3-kinase (PI3K). These mutations lead to hyperactivation of the PI3K/AKT/mTOR pathway, resulting in abnormal cellular growth and proliferation. The spectrum includes conditions such as CLOVES syndrome (Congenital Lipomatous Overgrowth, Vascular malformations, Epidermal nevi, and Skeletal anomalies), Megalencephaly-Capillary Malformation syndrome (MCAP), and Fibroadipose Hyperplasia. These disorders are characterized by asymmetric overgrowth, vascular malformations, skeletal abnormalities, and connective tissue dysplasia.

The diagnosis of PIK3CA is established through histopathological analysis of biopsy samples obtained from the venolymphatic malformations and genetic testing confirming the presence of a somatic PIK3CA mutation, consistent with the clinical presentation of CLOVES syndrome.

**Fig. 1:**
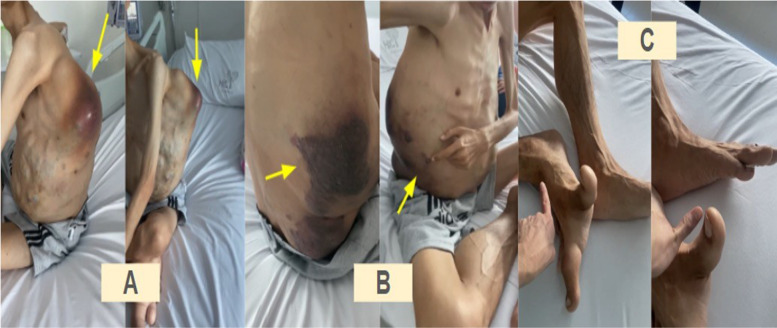
**A** Dorsolateral tumors. **B** Anterolateral abdominal tumors, ocher dermatitis. **C** Sandal gap deformity. Overgrowth of the lower limb extremities. Red (27%), white blood cells (9 mg/dL) and platelet count (3000 platelets/μL)

We present the case of a 32-year-old male patient with suspected PROS syndrome, clinically diagnosed as Congenital, Lipomatous, Overgrowth, Vascular malformations, Epidermal nevi, Spinal/skeletal anomalies and/or scoliosis (CLOVES) syndrome, characterized by multiple thoracoabdominal venolymphatic and arteriovenous malformations. The patient was admitted to the emergency department with bleeding from cystic lesions the left infrascapular region and severe thrombocytopenia. Biopsies were planned to confirm PIK3CA gene involvement and guide treatment initiation (Fig. 1A, B). The patient also exhibited macrodactyly, a hallmark feature of the syndrome (Fig. 1C).

**Fig. 2:**
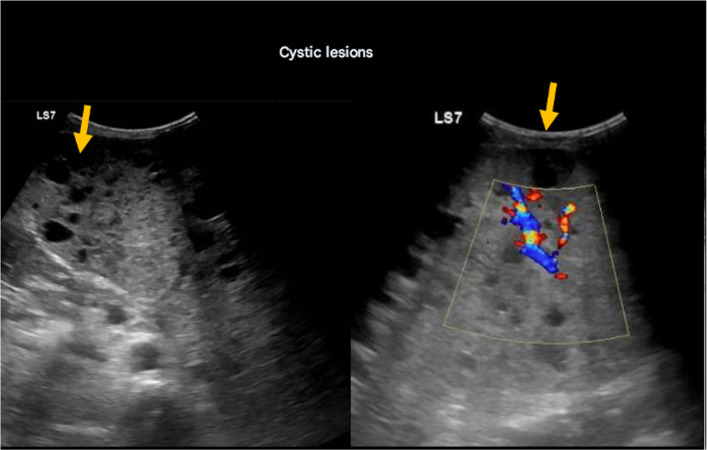
Ultrasound of spleen showing enlargement and multiple cystic lesions

The patient exhibited multiple clinical features of CLOVES syndrome, including a prominent thoracoabdominal mass, hypervascularized areas measuring up to 15 cm, macrodactyly, and inflammatory changes in the lesion. Ultrasound imaging of the splenic parenchyma revealed a large heterogeneous mass with cystic lesions (Fig. 2). The patient was hospitalized due to severe pancytopenia (3000 platelets/μL) and bleeding from cystic lesions, requiring urgent evaluation and management. Biopsies were required to confirm PIK3CA gene involvement and guided treatment.

**Fig. 3:**
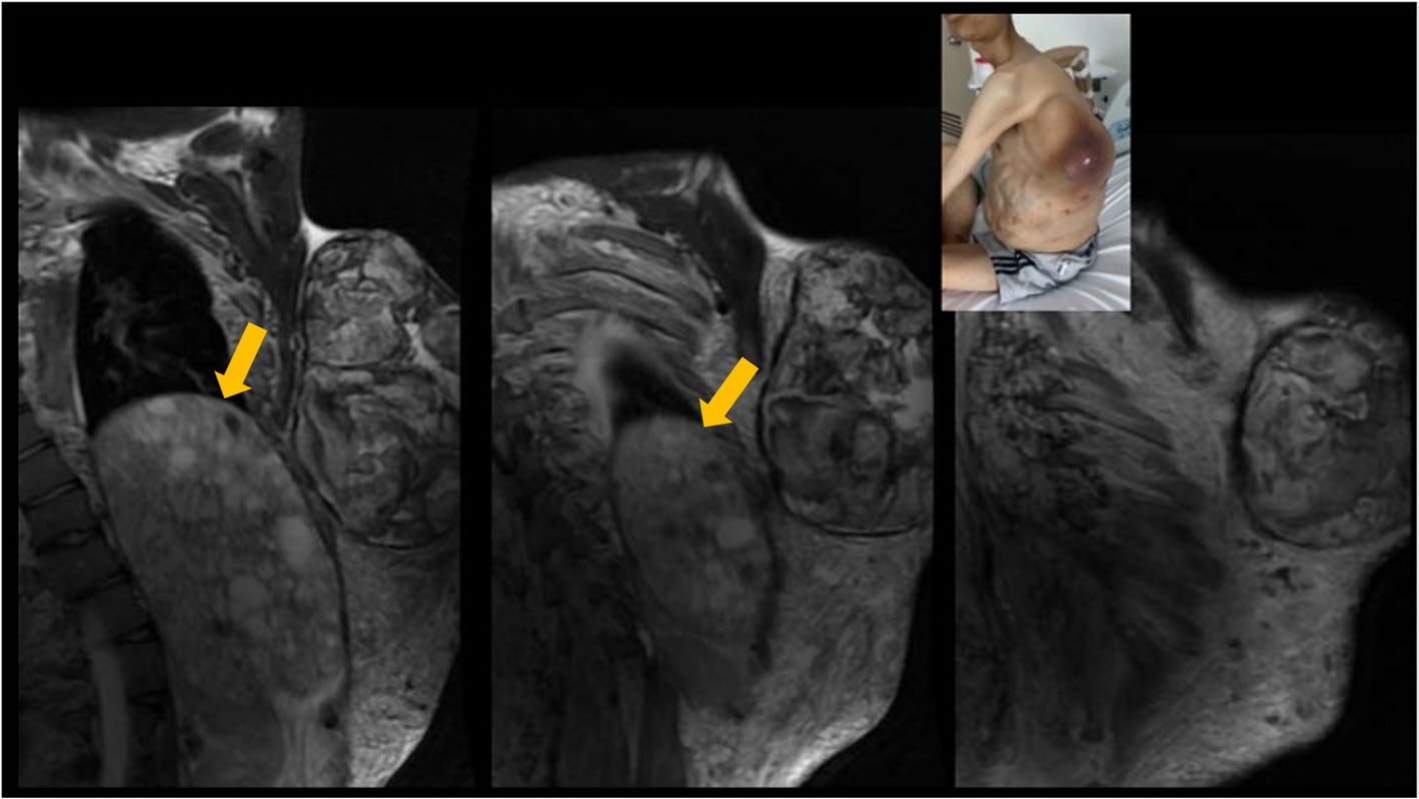
MR angiography of the thorax and abdomen. **A** Severe splenomegaly with pseudonodular tissue formation in the subcutaneous and left paravertebral tissue

**Fig. 4:**
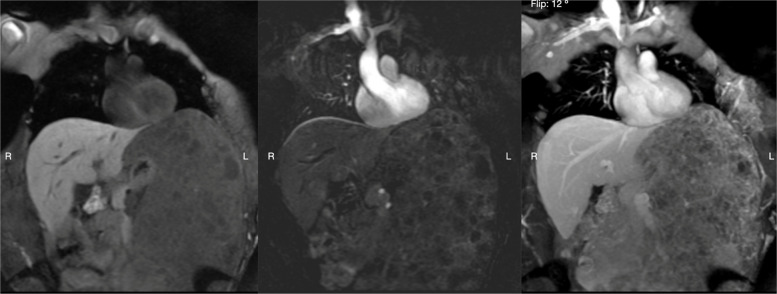
MR angiography of the thorax and abdomen: Vascularized lesion with progressive enhancement, possibly venolymphatic in nature, and the presence of multiple tubular images. MR angiography of the thorax and abdomen: The imaging revealed severe splenomegaly associated with multiple cystic lesions that did not enhance after contrast administration, consistent with multiple lymphatic malformations. Given the complexity of performing a biopsy in this patient’s clinical condition (severe thrombocytopenia), the procedure required intervention by an interventional radiologist. Additionally, it is important to note that the PICK3CA gene exhibits a high degree of mosaicism and has low detection sensitivity. Therefore, it has been demonstrated that the best site for biopsy is directly from the malformation, as this increases the likelihood of detecting the mutation

Subsequently, an MR angiography of the thorax and abdomen was performed, revealing voluminous venolymphatic malformations in the soft tissues of the dorsal and thoracoabdominal regions, extending towards the vertebral bodies and pelvis (Fig. 3), with an arteriovenous component observed by the presence of tubular images with hyperintense signals on T2 and hypointense on T1, showing enhancement during arterial and venous phases, indicative of vascular involvement (Fig. 4).

### Transvenous biopsy technique

Following aseptic preparation of the left intrascapular dorsal region and the administration of local anesthesia with 10 ml of 1% lidocaine (Ropsohn Therapeutics s.a.s, Bogotá, D.C., Colombia). Due to the patient’s severe thrombocytopenia, prophylactic platelet transfusion (2 units) was administered prior to the procedure to minimize bleeding risk. The target lesion and access vein were carefully selected using ultrasound imaging, with soft tissue ultrasound tracing identifying a subcutaneous drainage vein adjacent to the lesion avoiding arterial structures. Hydrodissection of the subcutaneous tissue was performed under ultrasound guidance with color Doppler using a 22-gauge spinal needle. A 0.9% saline solution (Baxter, Cali, Colombia) was injected to separate venous vascular structures from arterial ones, ensuring safe access to the target lesion. An 18 gauge Achiba needle (Cook Medical LLC, Bloomington, USA), was advanced to reach the dominant venous structure leading to the lesion to be biopsied. The biopsy needle was directed under ultrasound guidance to ensure precise targeting of the lesion. A Cope guidewire was then advanced coaxially, and the needle was exchanged for a Neff 6 Fr (Cook Medical LLC, Bloomington, USA) set. The Cope guidewire was replaced by a 0.035-inch diameter Teflon coated guidewire (Terumo Medical Corporation, NJ, USA), over which a 6 Fr dilator was advanced. The dilator was then exchanged for the outer cannula of a 17G TruGuide needle (BD, AZ, USA).Next, the Teflon guidewire was removed, and the inner stylet of the TruGuide needle was placed, allowing puncture of the solid lesion in the venolymphatic malformation under ultrasound guidance. Once the TruGuide needle was positioned inside the lesion, the stylet was removed, and a coaxial 18G Tru-Cut needle was advanced to obtain five tissue samples. Post-biopsy, the site was imaged using ultrasound to confirm hemostasis and rule out complications, the needle was then withdrawn and a hemostatic plug with Spongostan (J&J Med Tech, New Brunswick, USA) was applied.

**Fig. 5:**
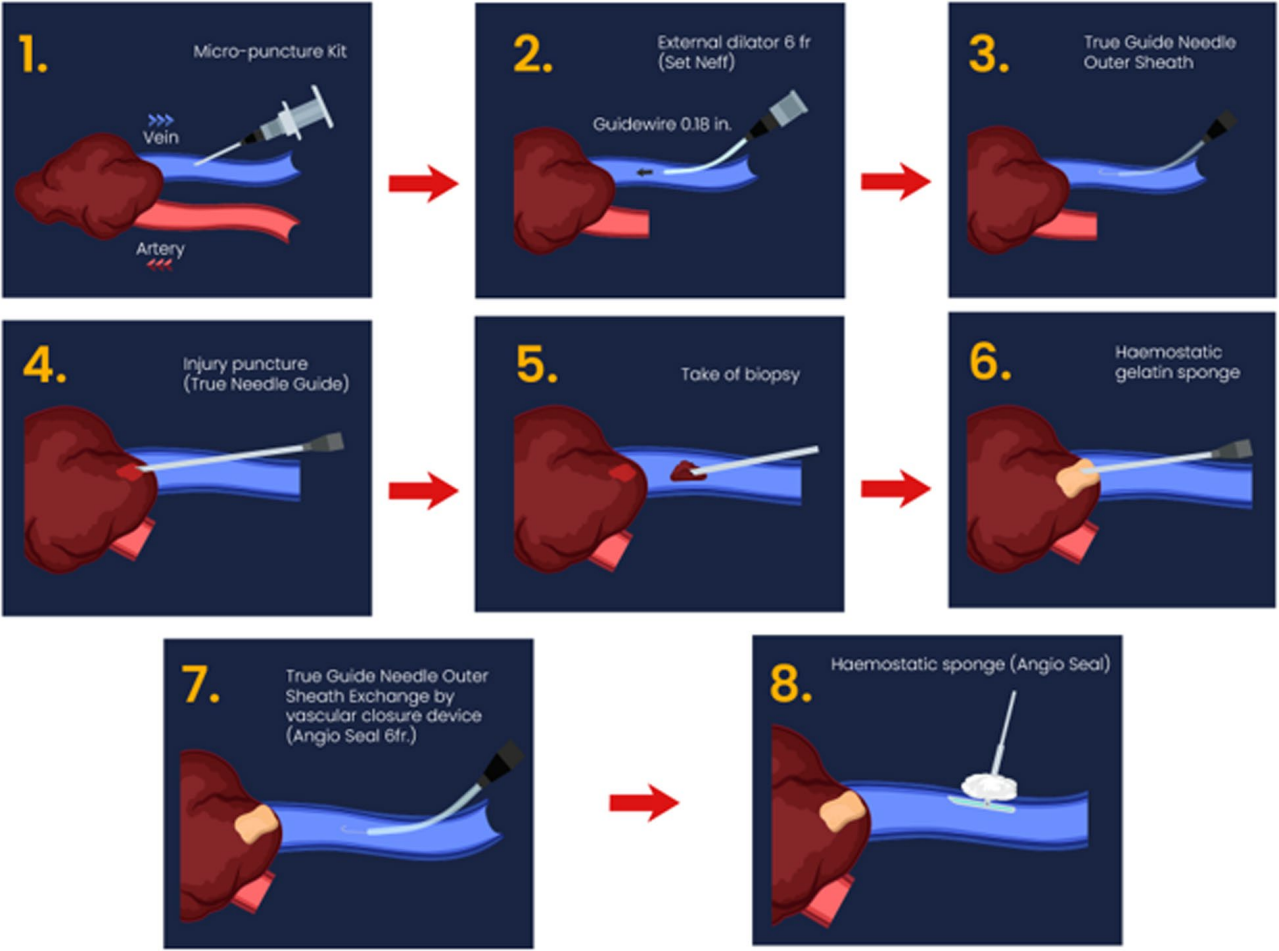
**A** Biopsy procedure illustration. **B** Biopsy procedure as seen on computed tomography

Subsequently, the Teflon guidewire was advanced through the outer cannula of the TruGuide needle to the external opening (outer sheath), ensuring that the guidewire remained within the intravascular space. A 6 Fr AngioSeal (Terumo Medical Corporation, NJ, USA), was advanced over the guidewire for vascular closure at the venous level. After removing the AngioSeal system, skin closure was performed using Histoacryl (B. Braun, Melsungen, Germany). Venography was not performed before or after biopsy (Fig. 5).

## Discussion

Percutaneous biopsy is considered the first line technique for evaluating abdominal lesions due to its broader accessibility, minimal invasiveness, and high diagnostic accuracy. However, it is contraindicated in patients at high risk of bleeding and/or in those with lesions close to complex structures due to the risk of perforation [6, 7].

In patients with severe thrombocytopenia, performing invasive procedures such as a biopsy can pose a significant challenge due to the high risk of hemorrhage. In such cases, conventional techniques like percutaneous biopsy may be contraindicated, prompting the need to explore safer alternatives [8]. In this context, transvenous biopsy emerges as a viable option, as it allows tissue sampling through the vein in a more direct manner. Any bleeding that may occur is contained within the venous system, where it is recirculated, preventing blood loss in the patient [9, 10].

The transvenous technique not only reduces the risk of bleeding but also employs closure systems that enhance patient safety, as demonstrated in this procedure. Two main closure systems are used: first, a hemostatic sealant like Spongostan (J&J Med Tech, New Brunswick, USA) is applied at the lesion site to promote coagulation and prevent bleeding. The second system, Angio-Seal (Terumo Medical Corporation, NJ, USA), is used to close the vascular access once the biopsy is completed, minimizing the risk of external bleeding. Both systems can be implemented with ultrasound guidance, improving procedural safety and allowing replication in various areas of the body, as long as there is access to a nearby vein at the desired biopsy site [11].

Finally, treatment without histopathological confirmation is often the approach taken in emergency scenarios where urgent decision-making is required, which can lead to inaccurate choices and alter the course of treatment. However, biopsy is not always indispensable if the diagnosis can be made through imaging, making it a viable alternative for patients with known risk factors, such as coagulation disorders, complex anatomies, or conditions requiring immediate management [12].

Transvenous biopsy represents a safe and appropriate alternative for obtaining tissue samples in patients with conditions that carry a high risk of bleeding and intraoperative complications, particularly in those with coagulation disorders, such as severe thrombocytopenia. Based on the results achieved with this technique in transjugular liver biopsies and transfemoral renal biopsies, it minimizes the risk of hemorrhage by accessing the venous system, which proves particularly beneficial. It is essential to continue training in this procedure and consistently evaluate its safety and efficacy in comparison to other techniques such as percutaneous biopsy and open biopsy.

## Conclusion

Percutaneous biopsy is the first-line technique for evaluating abdominal lesions due to its accessibility, minimal invasiveness, and high diagnostic accuracy. However, it is contraindicated in high-risk patients, such as those with severe thrombocytopenia or lesions near complex structures, due to the risk of bleeding and perforation. In such cases, transvenous biopsy emerges as a safer alternative, allowing tissue sampling through the venous system, where any bleeding is contained and recirculated, minimizing blood loss.

## Data Availability

Not applicable.
